# Sequential neuronal processing of number values, abstract decision, and action in the primate prefrontal cortex

**DOI:** 10.1371/journal.pbio.3002520

**Published:** 2024-02-16

**Authors:** Pooja Viswanathan, Anna M. Stein, Andreas Nieder

**Affiliations:** Animal Physiology, Institute of Neurobiology, University of Tuebingen, Tuebingen, Germany; Radboud Universiteit Donders Institute for Brain Cognition and Behaviour, NETHERLANDS

## Abstract

Decision-making requires processing of sensory information, comparing the gathered evidence to make a judgment, and performing the action to communicate it. How neuronal representations transform during this cascade of representations remains a matter of debate. Here, we studied the succession of neuronal representations in the primate prefrontal cortex (PFC). We trained monkeys to judge whether a pair of sequentially presented displays had the same number of items. We used a combination of single neuron and population-level analyses and discovered a sequential transformation of represented information with trial progression. While numerical values were initially represented with high precision and in conjunction with detailed information such as order, the decision was encoded in a low-dimensional subspace of neural activity. This decision encoding was invariant to both retrospective numerical values and prospective motor plans, representing only the binary judgment of “same number” versus “different number,” thus facilitating the generalization of decisions to novel number pairs. We conclude that this transformation of neuronal codes within the prefrontal cortex supports cognitive flexibility and generalizability of decisions to new conditions.

## Abstract 2

Decision-making needs evaluation of options, deliberation and action-planning. This study shows that neurons in the primate prefrontal cortex sequentially delineate these processes to separable components of number values, abstract decision and motor planning.

## Introduction

Making decisions involves collecting sensory evidence, careful deliberation of options, making a judgment, and the corresponding choice. When a judgment, such as “same” or “different,” is based on 2 sequential stimuli, sensory information must be first represented and stored. Then, a comparison can be performed, that, in turn, prompts the preparation and execution of a response. Thus, any decision is preceded by the representation of stimuli and followed by an action indicating the response. This sequence of events requires that the information carried by the neurons in a given brain area changes with task events which might prioritize one function over another. While aspects of sensory and working memory representations of individual stimuli [1–3], as well as the execution of motor actions are well explored [4,5], how neurons efficiently and flexibly represent the entire sequence of a decision-making process is not known. In particular, whether this sequence of events consists of dissociable processes remains a matter of debate.

In the field of perceptual decision-making, decisions are traditionally thought of as a plan of action or making a choice [6,7]. In this framework, the decision is equated with motor preparation. This viewpoint is backed by studies that demonstrate a strong connection between decisions and associated motor actions or choices [8]. In these studies, association areas in the brain have been said to represent many cognitive processes in terms of embodied motor actions [9]. But decisions may sometimes be made as a result of an action-independent process. Such a dissociation of the decision and associated action has also been reported during tasks that enforce a rule-based report of the decision, where it is considered an abstract decision [10–13]. In one recent study, the action-independent decision process has been conceived as a state of perception or memory sampling [14,15].

Despite its significance, the study of decision-making at the neuronal level without considering the subsequent action or choice has received limited attention. When the forthcoming action or choice remains unknown, the granularity of a decision becomes pertinent to investigate. We hypothesized that the neuronal representation of such a decision may encompass either the complete content of the perceptual options under consideration, a condensed version emphasizing the most relevant features, or an entirely abstract other category—an abstract decision. To investigate this, we developed a sequential numerical decision task. If the decision were dependent on the precise sequence of numbers, as outlined in the first hypothesis, we predicted number or sequence selectivity to be privileged over the selectivity for decision. In contrast, an abstract decision would be reflected by a separation of the decision representation both from the retrospective number or sequence representations and the prospective motor plan. We trained 2 monkeys to view 2 dot displays in a sequence and evaluate whether the numerical values of the 2 displays were the same or different. Only after a delay period and in conjunction with a delayed motor response rule could the monkeys plan and execute a response. This task sequence allowed us to investigate the contents and transformation of neuronal codes during the course of an abstract decision process in the primate prefrontal cortex (PFC).

## Results

**Fig 1:**
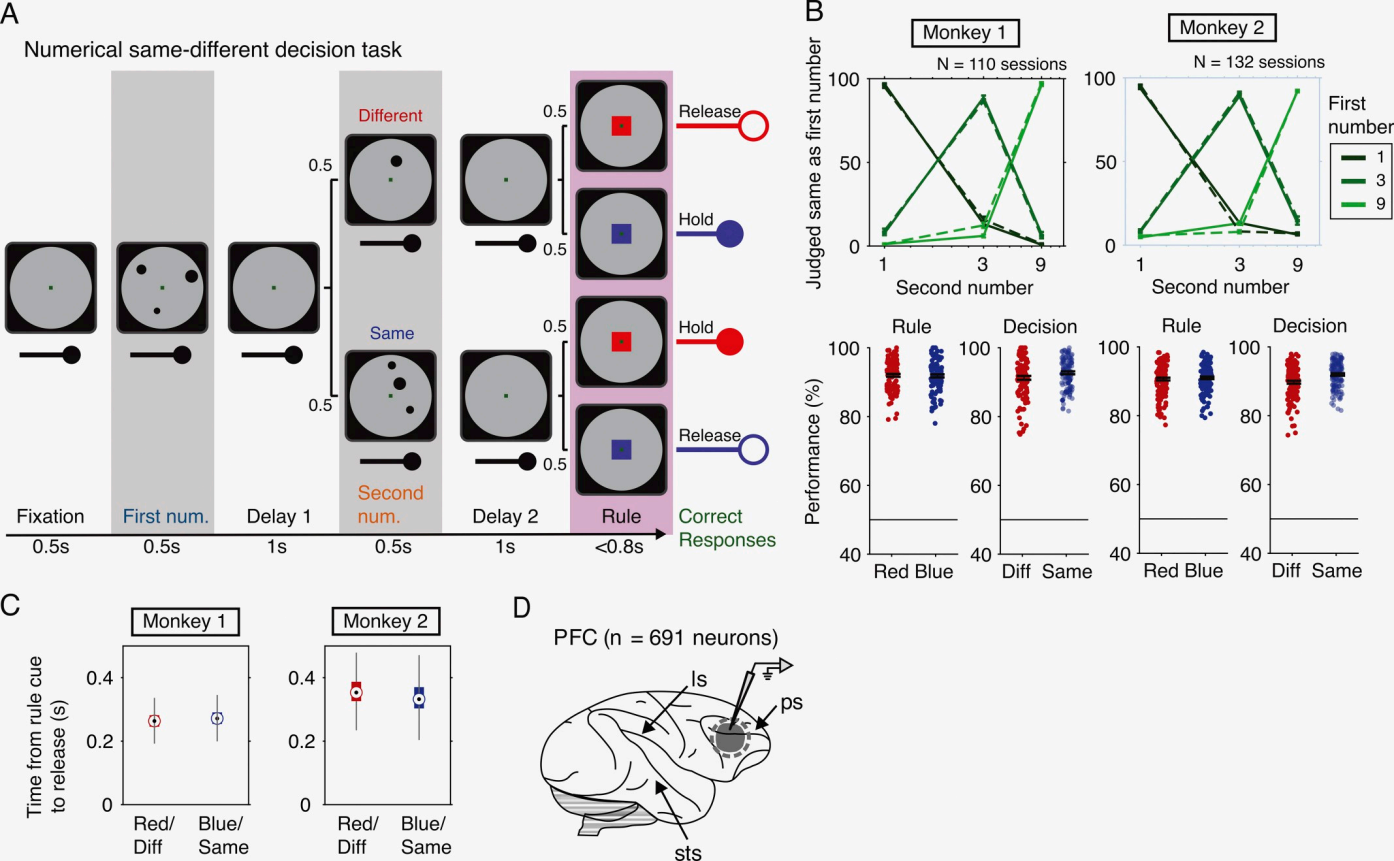
Numerical decision task and behavior. (**A**) The behavioral task is depicted by the screen displays and the response bar held by the monkey. Each trial begins with a fixation period requiring the monkey to look at the central fixation spot on the screen and hold the response bar (up to the rule period). Dot displays are shown in sequence, interspersed by a delay of 1 s. Following the second delay, the monkey can respond during the rule period by either releasing the bar or maintaining their hold. Top row shows the task contingencies for “different” numerosities (50% of the trials), bottom row for “same” numerosities. Correct combination of the “same/different” decisions and motor rules (orthogonalized for the 2 decisions) are rewarded by fluid. (**B**) Performance of monkeys M1 and M2. Top panel, percent correct responses to the 3 different numerosities. Solid lines for standard numerosity trials and dashed lines for control numerosity trials, mean ± SEM. Bottom panel, performance split by motor rule or by decision. Individual circles are behavioral sessions, with the black bar showing the mean ± SEM, bottom panel. (**C**) Reaction time is plotted as the time from rule cue onset to monkey’s release on trials when decision matched the motor rule. Box plots depict the median across trials with 25%–75% confidence intervals. (**D**) Recording area in the primate brain shown in gray, sagittal view with some anatomical landmarks. ls, lateral sulcus; sts, superior temporal sulcus; ps, principal sulcus. The data underlying this and all other figures is available at https://doi.org/10.6084/m9.figshare.25046987.

Disentangling decision-making from aspects of perception and action has posed a challenge. We developed a sequential numerical decision-making task to separate the processes in time (**Fig 1A**). Two monkeys learned to compare the number of items (“numerosity”) in 2 sequentially shown displays, to make a decision on whether the numerosity in both displays was same or different, and to respond according to an impending action rule presented at the end of the trial. The displays contained 1, 3, or 9 dots. To rule out the effects of lower level visual features that tend to co-vary with the numerosity of the displays, we used 2 sets of stimuli, one with randomly chosen radii and inter-dot distance (“standard”), another with controlled total area and inter-dot distance (“control”) (**S1 Fig**). The rule cue instructed the monkeys on the correct response in conjunction with the monkey’s previous decision on whether the dot displays contained the same number of dots or different (**Fig 1A**): If the numerosities of the first and second stimulus differed, a red rule cue required the monkey to release a response bar, whereas a blue rule cue required the monkey to maintain their hold of the bar. In contrast, if the numerosities matched, the rule contingencies were inverted. Trials were counter-balanced for decision, number of dots used in first or second position, stimulus protocol and rule cue.

### Behavior in numerical decision task

Both monkeys performed well above chance (i.e., 50% performance) in more than a hundred sessions per individual. Overall, discrimination of the 3 numerosities was close to perfect and performance (correct responses) of both monkeys to the 2 rule cues (red/blue) and the 2 decision options (same/different) was around 90% (**Fig 1B**). No significant difference of the effect of rule cue on performance in either monkey was observed (Monkey M1, *n* = 110 sessions: Z = 0.40, *P* = 0.6880; Monkey M2, *n* = 133 sessions: Z = −0.35, *P* = 0.73; Wilcoxon rank sum test). Monkey M2 shows a small effect of decision on performance, performing significantly better on “Same” decisions (Monkey M1: Z = −1.95, *P* = 0.0513; Monkey M2: Z = −5.36, *P* = 8.2345E-08). The reaction time for trials in which the response rule required an instant bar release differed significantly for both monkeys across decision and rule cue combinations (**Fig 1C**). Monkey M1 showed slightly lower times for “Red” cue and “Different” trials (median = 0.26 s) than for “Blue” cue and “Same” trials (median = 0.27 s, Z = −18.79, *P* = 7.7637E-79, Wilcoxon rank sum test). Monkey M2 showed longer times for “Red” cue and “Different” trials (median = 0.35 s) than “Blue” cue and “Same” trials (median = 0.33 s, Z = 34.99, *P* = 3.1E-268). Similarly, the stimulus protocol had a small effect on the monkey M2’s discrimination performance (Monkey M1, Z = 2.4889, *P* = 0.0128; Monkey M2, Z = −4.0537, *P* = 5.0419E-05). On reaction times, stimulus protocol had a small effect in M1, no effect in M2 (Monkey M1, Z = 4.1198, *P* = 3.791E-05; Monkey M2, Z = −0.7768, *P* = 0.4373). Overall, performance in both monkeys was comparable across numerosities and decisions.

### Encoding of numbers, decision, and action

While the monkeys performed this task, we recorded a total of 691 neurons (291 neurons in monkey 1; 400 neurons in monkey 2) from the dorsal bank of the principal sulcus across sessions (**Fig 1D**). As shown previously [16–19], neurons in the dorsolateral PFC were selective for the number of items, especially during stimulus presentation. We observed that single neurons showed selectivity to different task factors at various times across a single trial. We performed all the subsequent analyses on neuronal responses collected in sliding temporal windows across the trial.

**Fig 2:**
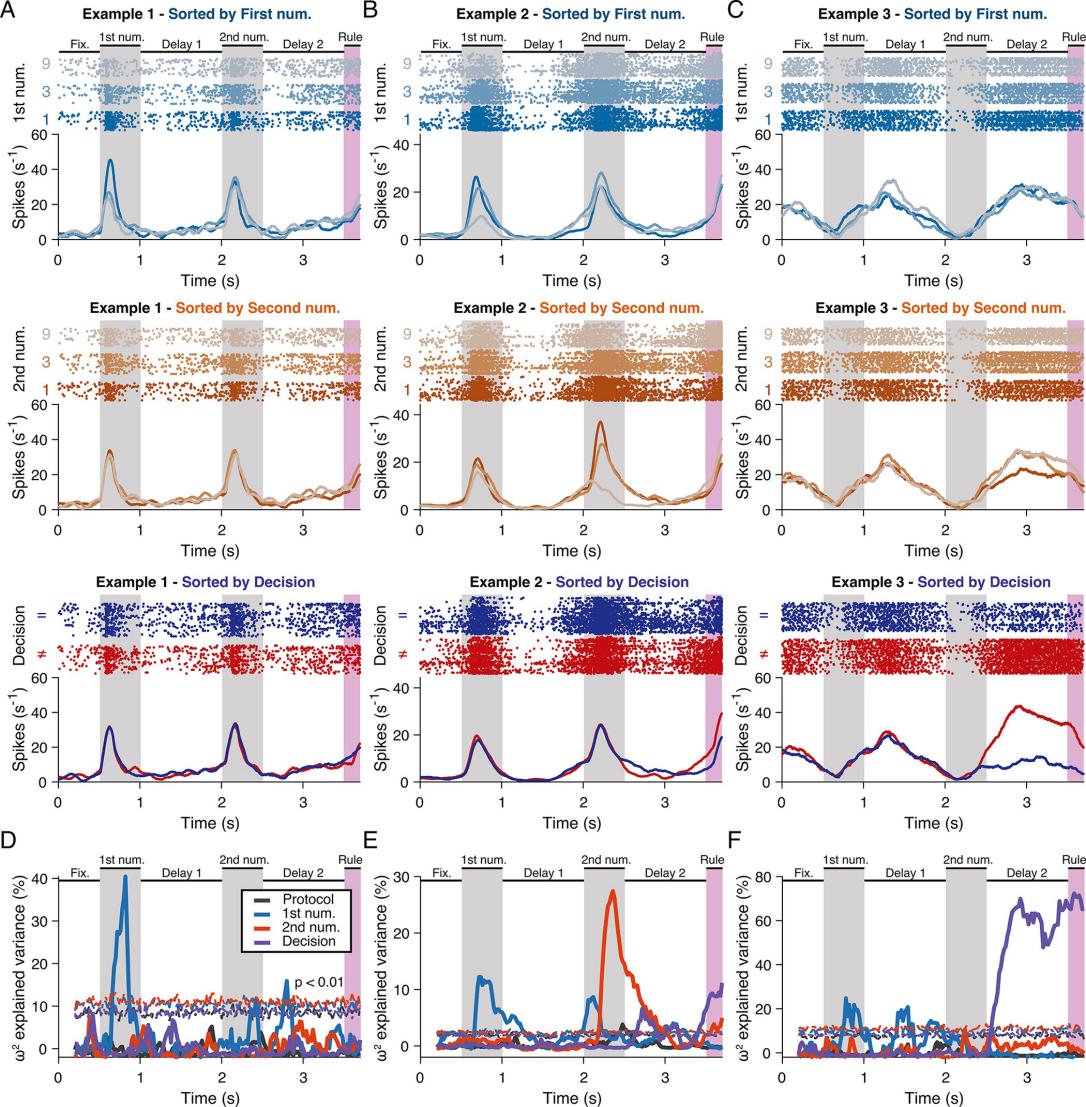
Single neurons in the prefrontal cortex show selectivity for task factors. (**A**) Example neuron 1 selective to numerosity in the first number period. The first column shows the same neuron’s responses sorted according to the first number (top), the second number (middle), and the decision (bottom). Each panel consists of a dot-raster histogram where each line is a trial, each dot is an action potential, and the corresponding spike-density histogram below showing time-resolved smoothed average firing rates. In each panel, the respective task factors, the numerosities in the first number period (top) and the second number period (middle), or the decision (bottom) are color coded. (**B**) Example neuron 2 selective to the first and second numerosity during each number presentation period. This neuron is also selective for the decision. Layout as in (**A**). (**C**) Example neuron 3 selective to the decision in the second delay period. Layout as in (**A**). (**D**) Information contained in the responses of example neuron 1 (**A**) about the 4 different task factors expressed as omega-squared percentage explained variance. The dotted lines indicate the 99th percentile of the effect size calculated from the distribution of values obtained from shuffled data. (**E**) Information contained in the responses of example neuron 2 (**B**). Layout as in (**D**). (**F**) Information contained in the responses of example neuron 3 (**C**). Layout as in (**D**). The data underlying this and all other figures is available at https://doi.org/10.6084/m9.figshare.25046987.

Some neurons were selective to numerosity in the first number period (**Fig 2A**), whereas other discriminated numerosity in both presentation periods (**Fig 2B**). Yet, other neurons were primarily selective in the delay periods (**Fig 2C**). Many neurons, however, were selective to more than one task factor, such as both the first and second number (**Fig 2B**), or both the first number and the decision (**Fig 2C**). We quantified for each neuron the effect size for stimulus protocol (as a control for visual parameters), first and second number, and decision by calculating the omega-squared, proportion of explained variance (PEV), a measure of information about the respective task factors contained in the neuronal activity (see Methods). Task factors such as the first number and/or the second number, for example, neurons 1 and 2, respectively, (**Fig 2D and 2E**), or the decision for example neuron 3 (**Fig 2F**) explained a large proportion of the variance in the trial-by-trial firing rates of single neurons, tested for each neuron against a distribution of PEV values calculated from shuffled labels for each task factor (*P* < 0.01). Only 13 neurons overall (2%) showed selectivity for the stimulus protocol, 2 neurons for rule cue, and 1 neuron for the monkeys’ action. The temporal PEV-analysis revealed single neurons’ selectivity to multiple task factors; PFC neurons showed mixed selectivity for both numbers and decision (**S2 Fig**). However, many neurons were selective purely for one factor across time: 58% (68/117) of decision-selective neurons were only selective for decision, whereas 34% (39/115) and 34% (28/83) of number-selective neurons were selective exclusively for the first and second number, respectively. Thirty-five percent (51/147) of neurons were selective for numbers regardless of their order. A large percentage (61%) of these neurons maintained their selectivity across order, i.e., preferred the same number during first and second number presentation (31/51). Thirty-nine percent (20/51) neurons changed their preference.

### Decoding of task factors across time

**Fig 3:**
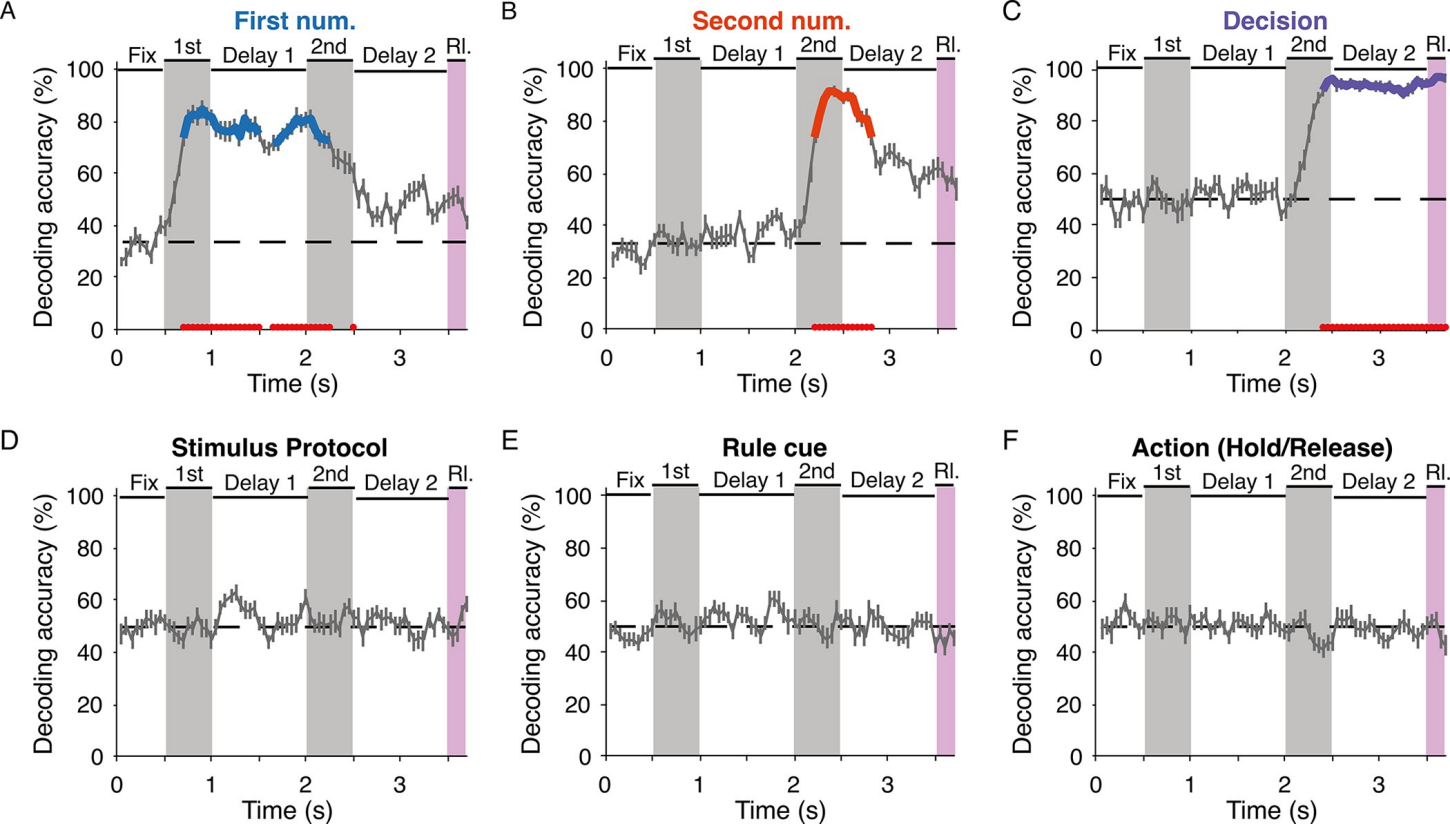
Relevant task factors can be decoded from the neuronal population in PFC, but not the irrelevant stimulus features nor the impending rule cues or actions. (**A**) SVM-classifier decoding accuracy for factor “first number” (numerosities 1, 3, 9) plotted against time (mean ± SEM across 20 runs). Dashed horizontal line indicates chance level for the number of classes in each decoder. The true accuracy of decoding on held-out test trials is tested against the 99th percentile of 1,000 permutations of decoding from classifiers trained on shuffled trials. Thickened color lines and red circles plotted along the time axis indicate significant decoding (*P* < 0.01). (**B**) Decoding accuracy for factor “second number” (numerosities 1, 3, 9). Same layout as in (**A**). (**C**) Decoding accuracy for factor “stimulus protocol” (std/control). Same layout as in (**A**). (**D**) Decoding accuracy for the factor “decision” (same/different). Same layout as in (**A**). (**E**) Decoding accuracy for factor “rule cue” (red/blue). Same layout as in (**A**). (**F**) Decoding accuracy for factor “action” (hold/release). Same layout as in (**A**). The data underlying this and all other figures is available at https://doi.org/10.6084/m9.figshare.25046987. PFC, prefrontal cortex; SVM, support vector machine.

Across the population, we evaluated how different task factors were organized in time by training support vector machines (SVMs) to decode them. We extracted a population with a minimum number of trials per level for each factor (*n* = 537 neurons). With this pseudo-population, we asked whether perceptual, cognitive, and motor factors were separable in a time-resolved way. We performed 20 runs of decoding for each task factor by resampling training and test trials to get an average estimate of the pseudo-population. For each factor and run, an SVM was trained on 10 training trials per level with 5-fold cross-validation and its accuracy was tested on 10 held-out trials. The accuracy of decoding was statistically tested against 1,000 permutations of decoding performed with shuffled training trials (*P* < 0.01). Based on the decoding accuracy, we found that the population initially represented the first number (**Fig 3A**) and the second number (**Fig 3B**). The classifier was not able to decode the stimulus protocols we used to control for the area and density of the dot displays, indicating that the neuronal population did not contain information on purely visual and nonnumerical parameters (**Fig 3C**). Toward the end of the presentation of the second numerosity, the decision (“same” versus “different”) could be decoded with almost 100% accuracy (**Fig 3D**). In contrast, the impending action rule cue (“red” or “blue”) (**Fig 3E**) and the subsequent action (“hold” versus “release”) could not be decoded (**Fig 3F**). This indicates that only the task factors relevant for successful task completion were encoded by the neurons and that the abstract decision rather than rule cue-related and action-related information was encoded by the neuron population.

### Sequential transfer of information

**Fig 4:**
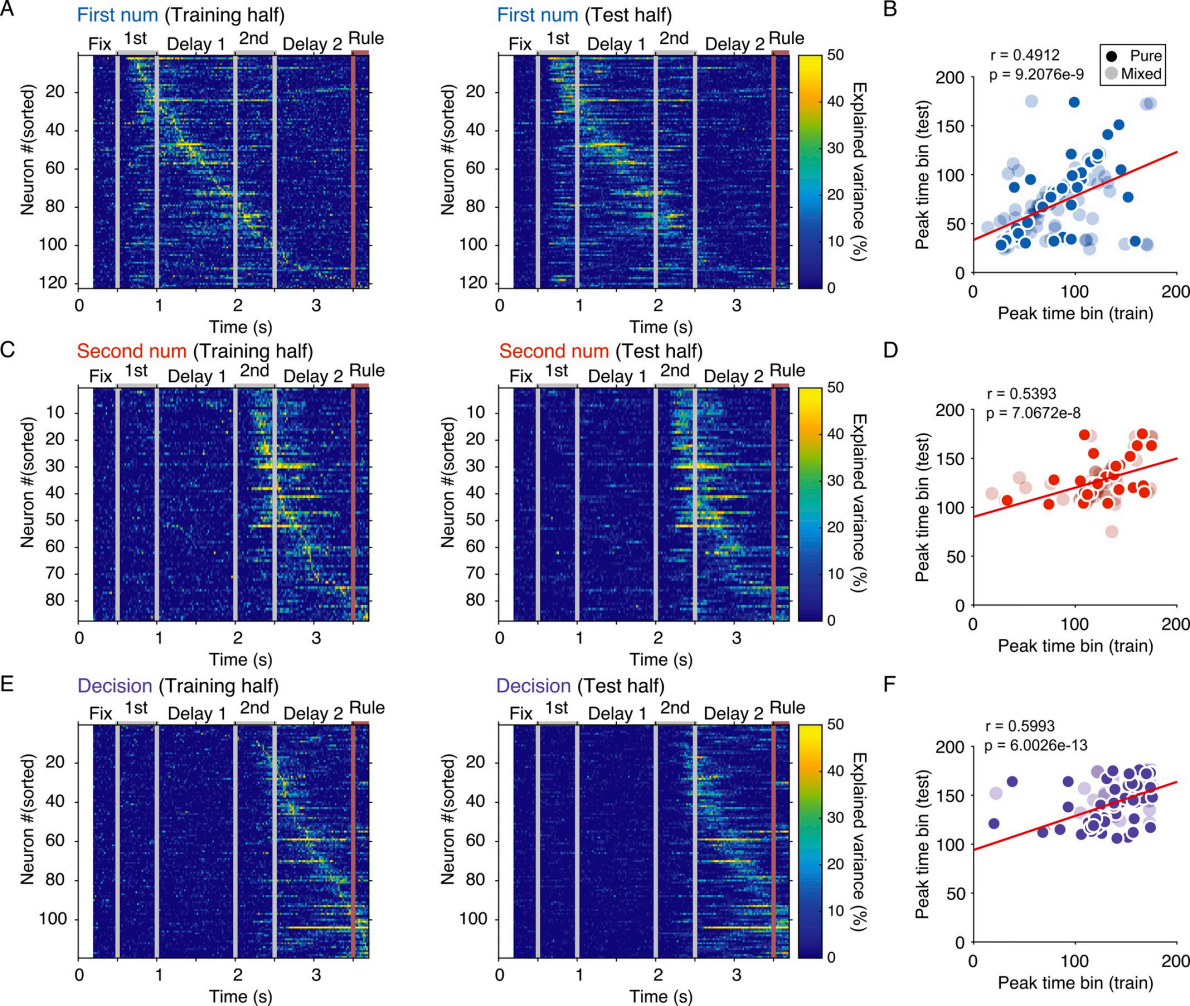
Neuronal selectivity is sequentially tiled. (**A**) Omega-squared variance explained for first number calculated from half the trials for neurons selective for first number, ordered by peak effect size, left panel. On the right panel, the same neuron order is imposed on effect size calculated from the testing half of trials. (**B**) The peak time from training half is correlated with peak time from testing half, individual neurons with a significant effect for first number are plotted. (**C**) Sequential order for neurons selective for second number. Same layout as in (**A**). (**D**) Peak effect size time bins for neurons selective to second number. Same layout as in (**B**). (**E**) Sequential order for neurons selective for decision. Same layout as in (**A**). (**F**) Peak effect size time bins for neurons selective to decision. Same layout as in (**B**). The data underlying this and all other figures is available at https://doi.org/10.6084/m9.figshare.25046987.

**Fig 5:**
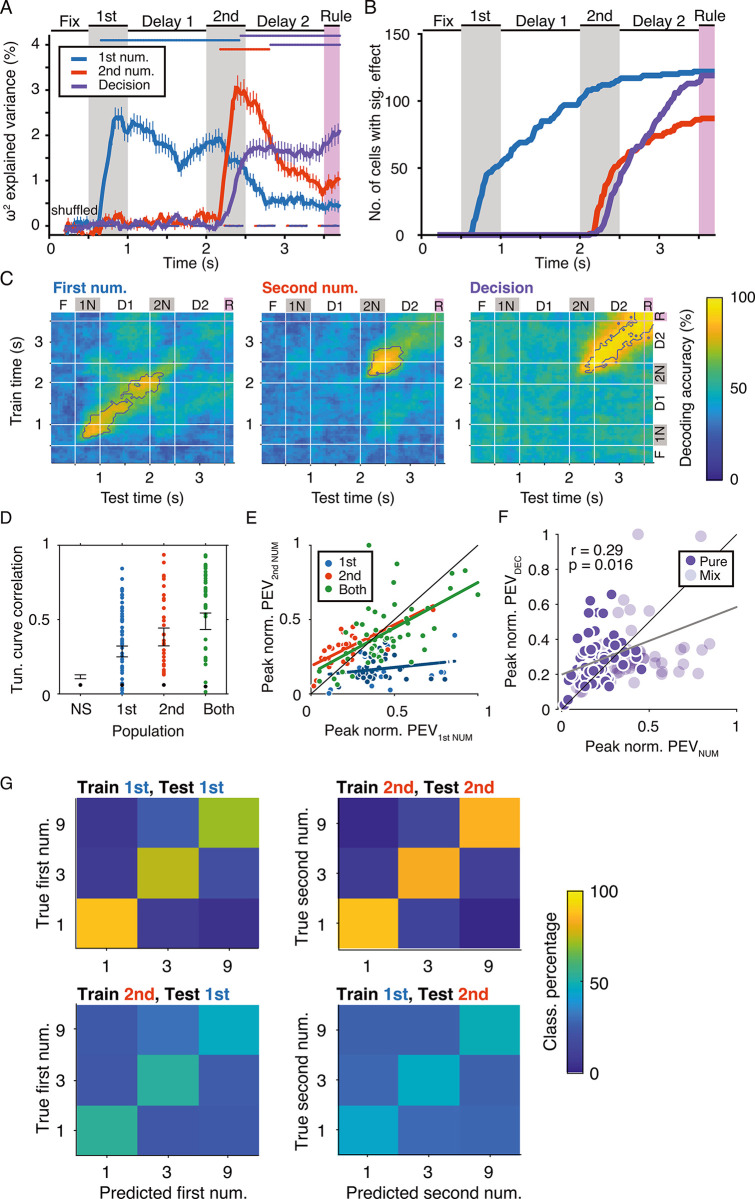
Sequential and dynamic tiling of neuronal activity in PFC. (**A**) Omega-squared percentage explained variance (PEV) across time and population for factors “first number,” “second number,” and “decision.” Colored lines represent mean ± SEM PEV across all neurons for each task factor. Dashed lines (overlapping) are averages across shuffled data for all neurons. Colored bars near the top indicate significant differences between first num and decision, second num and decision for each time bin, Wilcoxon rank sum (*P* < 0.01). (**B**) Cumulative distribution of neurons reaching significant coding of a task factor. Significance determined for each neuron separately against the 99th percentile of omega-squared PEV distribution obtained from 1,000 shuffles each. (**C**) Cross-temporal decoding of “first number” (left), “second number” (middle), and “decision” (right) depicted in confusion matrices. Black outlines in the confusion matrices indicate significant decoding accuracies. (**D**) Cross-correlation coefficients between single-neuron tuning curves for non-number selective neurons (NS, *n* = 570) and each neuron with selectivity for first number (1st, *n* = 40), second number (2nd, *n* = 51), and both (*n* = 30). Values for individual cells are plotted in color, their mean ± SEM in black. Black dots close to the bottom indicate the mean + 3 SD cross-correlation calculated from shuffled data. (**E**) Correlation of effect sizes (PEV) for information about first numerosity and second numerosity for neurons selective to numerosity, for first number only (cyan), second number only (orange), selective for both numbers (green). Gray (nonselective neurons not plotted individually, r = 0.3883, *P* = 5.9099E-22), orange (r = 0.4475, *P* = 0.0011), and green (r = 0.491, *P* = 0.0064) full lines indicate significant correlation of peak effect size for “first number” and “second number” of different populations. Dashed line indicates nonsignificant correlation in neurons selective for first number only (r = 0.3788, *P* = 0.0165). (**F**) Correlation of effect sizes (PEV) for information on task factors “number” and “decision” in decision-selective neurons. Pure decision-selective neurons show a weak correlation. Decision-selective neurons with mixed effects also show a weak correlation (r = 0.3545, *P* = 0.0111). (**G**) Confusion matrices for decoding scores from decoders trained and tested on numerosity. Top left: Trained and tested on the “first number”; top right: trained and tested on “second number”; bottom left: trained on “second number,” tested on “first number”; bottom right: trained on “first number,” tested on “second number.” The data underlying this and all other figures is available at https://doi.org/10.6084/m9.figshare.25046987. PEV, proportion of explained variance; PFC, prefrontal cortex.

We observed, however, that the decoding of numbers was not sustained till the appearance of the rule cue (**Fig 3A and 3B**) when the corresponding action could be performed. Instead, the neuronal population held information about the numbers primarily during the presentation periods. This was confirmed using PEV as a measure of single-neuron information content. We calculated PEV for each task factor: first number, second number, and decision using 50% of the trials for each neuron. We ordered the neurons according to the latency of their peak significant encoding (**Fig 4**) in the 50% of the trials used for training and observed that neurons encoded task factors sequentially. Applying the neuron order from the training trials to the PEV values calculated from the remaining 50% test trials reproduced the sequential patterns across single neurons, resulting in significant correlation between the peak encoding time of the training and test trial halves for each task factor. The selectivity for each task factor at the single neuron level (**Fig 4**), and when taken together as a population (**Fig 5A**), indicates that task information was sequential and dynamic. Once the number of neurons selective to the first and second numerosities saturated, more and more neurons became selective to the decision (**Fig 5B**). We verified this finding in the population with a cross-temporal decoding analysis performed on the neuronal population (**Fig 5C**). Cross-temporal decoding by SVMs trained at a specific time period and tested at another showed significant performance accuracy mainly along the diagonals for each factor and temporally confined to the respective task periods of the first number (**Fig 5C**, left), second number (**Fig 5C**, middle), and decision (**Fig 5C**, right).

We separately investigated how selectivity for number changed with time by comparing (i.e., cross-correlating) the neurons’ tuning response profiles (**S3A and S3B Fig**). Number tuning curves were significantly correlated across number presentation periods and most correlated for neurons that exhibited selectivity during both presentation periods (**Fig 5D**). We also saw significant correlation for the effect size for number, such that neurons that were selective for both number periods carried similar levels of information about the numerosities presented in the 2 number periods (r = 0.58, *P* = 1.0125E-05) (**Fig 5E**). Neurons selective to the second numerosity only had some information about the first numerosity (r = 0.62, *P* = 1.9539E-04). These comparisons indicated that rather than changing their responses entirely with time, single neurons carried similar kinds and levels of information. In addition, a weak correlation existed between the peak PEV-effect size for numerical information (peak across first number and second number) and decision information for decision-selective neurons; decision-selective neurons typically had higher effect size (PEV) values for the factor, decision (**Fig 5F**). Neurons selective for the second number had correlated effect sizes for number and decision (**S3C Fig**). Overall, these analyses showed that a subpopulation of number-selective neurons maintained their selectivity for number across time. Decision-selective neurons also carried numerical information.

Despite the high degree of correlation in the response profiles and numerical effect sizes in single neurons, significant decoding of numerical values was only achieved from the population when the decoders were trained on the same order as they were tested (**Fig 5G**). High classification performance was seen if classifiers were trained and tested on the first number period, or trained and tested on the second number period (**Fig 5G**, top row). However, when classifiers were trained on the second number and tested on the first number, or vice versa, decoding did not reach significance (**Fig 5G**). The neuronal population seemed to carry information about whether the numerosity display was the first or the second numerosity that prevented cross-order number decoding, such that, for example, 1–3 trials drew a different response than 3–1 trials. The accuracy of decoding showed a response profile similar to that in single neurons (**S3A and S3B Fig**) with more errors occurring for numbers of higher magnitude (numerical magnitude effect) and when the numbers being compared were closer together (numerical distance effect). In cross-order decoding, the scores followed the same pattern during the presentation periods. At the population level, thus, the sequence of numerosity mattered, and at any given time in the trial, only 1 numerosity (either the first or the second) dominated.

**Fig 6:**
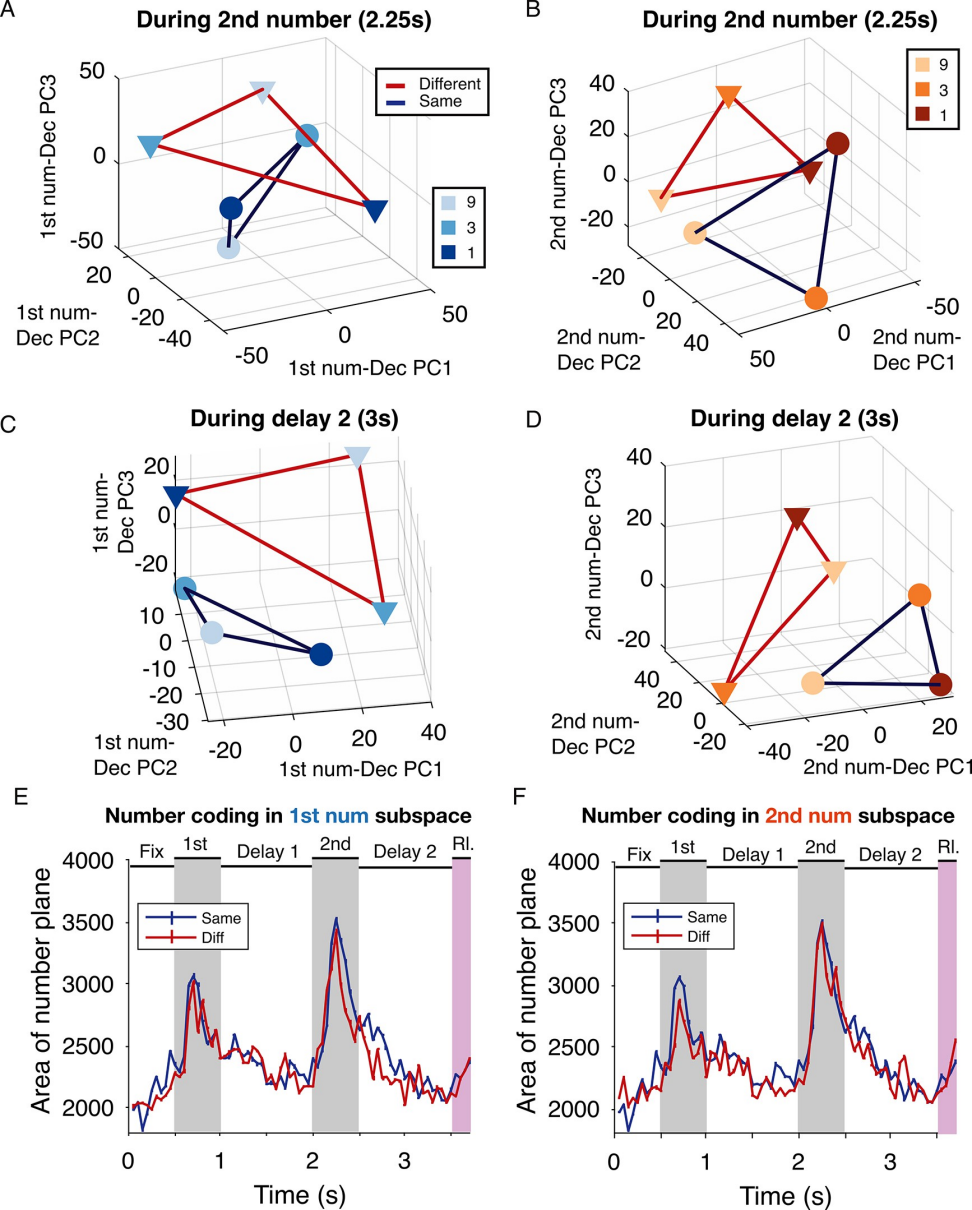
Number coding in the number-decision subspace is strongest during number presentation. (**A**) Neuronal population responses projected into a reduced dimensional subspace defined by the first 3 PCs of “1st number-decision” pairs. Individual numbers form the vertices of the plane for each decision (blue: same, red: different). Neural activity in this subspace is captured during the presentation of second number (2.25 s from start of trial). (**B**) Neuronal population responses projected into the subspace defined by the second number-decision pair captured during the second number period. (**C**) Same as (**A**) but captured late in the second delay (3 s from start of trial). (**D**) Same as (**B**) but plotted late in the second delay. (**E**) The area spanned by plane in the first number-decision subspace is plotted across time, mean ± SEM. The area is maximal during number presentation and returns to baseline at all other times. (**F**) Area spanned by plane in the second number-decision subspace. The data underlying this and all other figures is available at https://doi.org/10.6084/m9.figshare.25046987. PC, principal component.

All the analyses thus far showed that there existed a categorical representation of decision, separate from a simple representation of numbers and from the action, which could not be planned until the rule cue was presented. To better understand how the number representations gave rise to a separate categorical representation of decision, we performed a stepwise dimensionality reduction [20]. The time period from the second number presentation to the end of the second delay was indicative of such a transformation in the neuronal population. Whereas the first number must be maintained in working memory to be compared with the second number, the second number could be more directly manipulated and compared to give rise to the decision of same or different. The populations selective to the first and second number may therefore be differentially involved in the decision process. Thus, we collapsed neuronal activity onto a subspace created by the combination of the first number and decision (**Fig 6A**). The projection for “same” trials involving the numbers 1, 3, or 9 in the first presentation period formed a plane. The area defined by the vertices of such a triangle in the subspace captured the strength of number coding during “same” trials. Likewise, for “different” trials, a similar plane was observed. At this time during the trial, another subspace could be defined by the combination of the second number and decision (**Fig 6B**). During the second delay, we observed that the planes were much better separated in both subspaces and become more orthogonal (**Fig 6C and 6D**). We quantified the strength of number coding in both subspaces, under both decision regimes (**Fig 6E and 6F**). The numbers were best coded during the number presentation periods (gray shaded areas) and least well during the delays.

### Abstraction of the decision process

**Fig 7:**
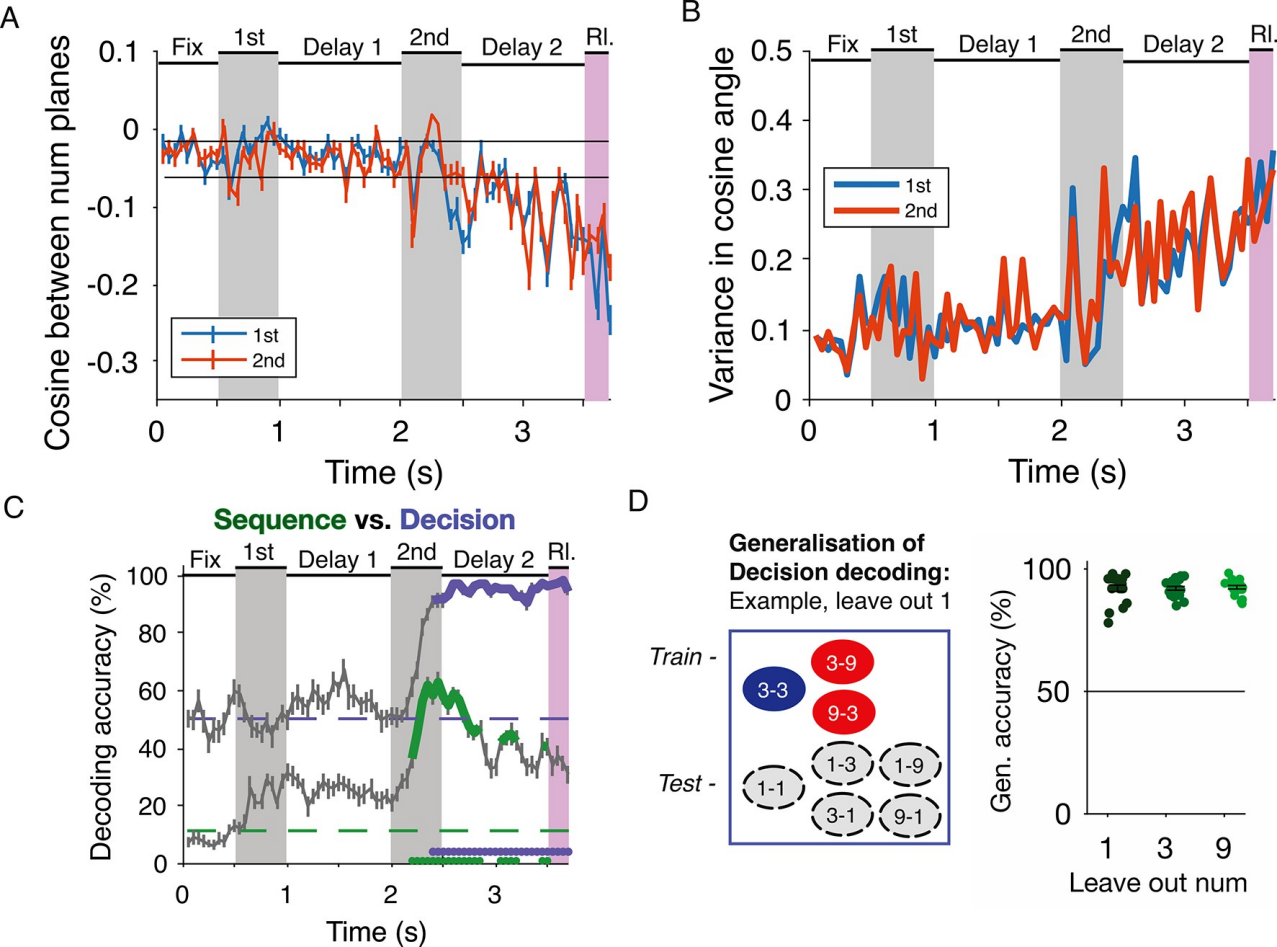
The decision subspace is largely abstract and invariant to number representations. (**A**) Cosine of the angle between the 2 planes over time. Mean ± SEM across runs is shown. Lower numbers indicate smaller difference. Black horizontal lines show the mean + 3 SD around the cosine from before the second number presentation. (**B**) Variance in the cosine angle across time increases after the second number period. (**C**) Performance of the sequence decoder across a trial. Significant performance (*P* < 0.01) of the sequence decoder is shown in green, compared to decision decoding for the same population in violet. Chance levels for each are shown as dashed horizontal lines. (**D**) Generalization of decision decoding across numbers tested by leaving out 1 number at a time. Schematic shows the combinations used for training and testing during Leave out 1. Each dot on the right is a run of decoding, mean across runs ± SEM in black. Chance decoding is 50%. The data underlying this and all other figures is available at https://doi.org/10.6084/m9.figshare.25046987.

Across time, this separability between the planes is captured by calculating the angle between them in each subspace, which begins to change after the second number is presented and continues to decrease in the late decision period (**Fig 7A**) as the planes get closer to orthogonal. The variance in the angle across runs of this analysis increases after the second number is presented (**Fig 7B**), illustrating that the variance in the neuronal population across trials is also maximal after the second number is presented, thus, setting off the transformation in neural space. These analyses together indicate that the neuronal population selective to the first numerosity and that selective to the second numerosity are similarly transformed by the decision process, albeit at slightly different times. In both subspaces, the decision process resulted in a loss of information about number.

This transformation of neuronal representations in the second delay period from number representation to a more abstract decision state is reflected in the decoding performance of SVM classifiers. In a new set of decoding tests performed on the neuronal activity, we tested the generalization of decision across numbers. First, we defined a pseudo-population (*n* = 368) with enough trials per number sequence (1–1, 1–3, 1–9, and so on) to classify the precise sequence of numbers. We sampled 3 training trials per sequence to train 20 runs of SVM classifiers and tested them to predict the precise sequence of numbers presented on 10 held-out test trials. Similar to the decoding profiles observed for individual numbers in the full population (**Fig 3**), the sequence decoder built with the responses of this pseudo-population performed best during the presentation of the second number, whereas the decision decoder of the same pseudo-population performed best after the second number has disappeared in the delay 2 (**Fig 7C**). The decision decoders were also trained with 3 training trials per decision and tested on 10 held-out test trials each time. The decoding performance was statistically tested against the 99th percentile of the distribution of accuracy from permuted training trials. Next, we returned to the full population (*n* = 537), to further test the abstractness of the decision representation across different number pairings, we tested if the decision decoder could generalize across numbers. We trained SVM classifiers to classify “same”-“different” decisions (5 training trials per decision) based on trials with combinations of 2 of the numbers while leaving out the third number at a time (**Fig 7D**). Then, we tested the accuracy of decoding on 10 held-out test trials involving the third unseen number. Across 20 runs of each classifier, the generalization accuracy for all numerosities was well above chance and the population achieved significant decoding such that the decision decoders that had learned to classify decisions based on 2 numerosities could generalize to those based on a third left-out number. Both these decoding tests illustrated that the decision process in the neuronal population was abstracted from the sequence of numbers as well as the individual numerosities in the late part of the second delay.

## Discussion

We demonstrate here that an abstract numerical comparison decision can be read out from the primate PFC prior to an intent to act. Using a sequential numerical decision-making task and a combination of single-neuron and population-level analyses, we observed a sequential transformation of neuronal information from a rich multifactor period to an abstract decision stage. While numerical values were represented with high precision and in conjunction with detailed information like order, decision was represented in a low-dimensional subspace that was number-invariant. At the final transformation, the binary “same/different” decision could be generalized to novel number pairs.

### Pure and mixed selectivity in the prefrontal cortex

Our first main observation is the presence of selective neurons for number and decision in the dorsolateral prefrontal cortex (dlPFC). We found neurons selective to numbers and decision in various combinations, as well as neurons exclusively selective to number, number order (only the first number or second number), or decision. The observation of mixed selective neurons together with more traditionally, selective single neurons in the dlPFC confirms previous findings [16,18,19,21–25]. While strongly selective single neurons could be said to form important network nodes engaging in complex computations or processing, mixed selective neurons are thought to expand the range of possible computations [26,27]. Our results align with the notion that mixed selective neurons increase the dimensionality of representations [28]. In particular, mixed selective neurons support reliable information transfer and are far more resistant to errors than pure selective neurons [29]. Not only does this allow the efficient use of neural resources by representing a wide range of information within a single neuron, but also the existence of mixed selectivity in the population enhances the capacity of flexible and context-dependent computations [30]. More recently, mixed selectivity has been reported to support high-dimensional geometry at the population level [31], which in turn facilitates cognitive flexibility.

### Dynamic coding in decision-making

Our results demonstrate that population-level decoding can accurately extract information about numbers and decisions from the neural activity. The successful decoding is consistent with the idea of dynamic coding, where information is represented in a distributed manner across the population, and the combination of activity patterns allows for the representation of complex and abstract concepts [32–35]. In line with this, previous research has shown that dynamic coding facilitates the flexible representation of decision variables in multiple brain regions [36,37]. The observed tiling of sequential activity suggests that the PFC engages in the temporal organization of neural activity, where the sequence of events in the task is reflected in the sequential firing of neurons. As has been widely reported, recurrent connectivity in the PFC integrates and transforms the information as it progresses through different stages of the decision-making process [38–40]. Recurrent neural networks can generate temporal patterns of activity that correspond to the flow of information in time [41].

In some recent studies, particularly in the PFC, a stable subspace has been reported to exist. Targeted dimensionality reduction produces a lower-dimensional stable subspace wherein working memory contents are faithfully represented for a longer duration [42–44]. This approach is perhaps ideal for the maintenance of information in the circuits during ongoing behavior. While we did not investigate the existence of a stable subspace in our population, we would expect that a sequential task requires more dynamic transfer of information for ready manipulation rather than maintenance. The same recurrent connectivity has been implicated in the maintenance of these stable subspaces [44]. It remains an open question how recurrent connectivity in the PFC facilitates these seemingly opposite requirements during complex behavior.

Our findings also confirm that abstract decisions are encoded in PFC in the absence of action-related activity. Since we trained the monkeys to withhold any action until the rule could be presented and motivate the correct response in association with a decision, we anticipated that any decision-related activity would be abstracted from action. A further transformation would be required after the rule cue was presented to select the appropriate action. Such a transformation could well happen within the prefrontal network we studied or other downstream areas [45–47]. Other studies using related task designs to separate the action selection and perceptual components from the decision-making process report similar report-independent decision correlates [10,48–51]. Our results can be contrasted readily against decision-making tasks where a choice or action can be prepared during a delay, and areas in the frontal cortex show strong correlations with the choice [5,52]. Action-independent correlates of decision or choice have been reported in other fields where the goodness or value processes are maintained in neural activity [53–55].

### Use of subspaces or transformations to disentangle task features

We found that, concerning number representation, the neuronal population even represented details such as the order and sequence of numbers. We interpret this as the monkeys using the first numerosity as a reference value and the second numerosity as a comparison value. This indicates that the representation of numbers is not reduced or abstracted as long as the decision on “same” versus “different” is pending. This is in contrast to the abstracted decision representation. One reason for this could be that the abstraction itself is a computation performed with the goal of future association [56,57]. In this study, since the decision is useful only in association with the action rule, the abstraction serves to reduce the dimensionality of information. The low-dimensional subspace can then be further transformed by the action rule to readout the current action. Such transformations are akin to those found during attentional manipulations and protection of working memory contents from distractors [20,58,59]. Another possibility is that the decision can be viewed as an abstraction of the number sequence itself. Once the neural activity moves into the decision subspace, it no longer requires the identity or the order of those numbers. This might be why the subspaces are not orthogonal to each other. This allows for interaction between the perceptual contents and the decision content. This might also facilitate communication with downstream areas [60] for integration with the rule cue or tracking of the task structure itself [61,62].

## Materials and methods

### Experimental model and subject details

Two adult male rhesus monkeys (*Macaca mulatta*), monkey 1 and monkey 2 served as experimental subjects for this study. They weighed between 6.5 and 8 kg in the duration of these experiments. Monkeys were socially housed under a 12/12-h light-dark cycle. They were provided with environmental enrichment for climbing and foraging. Primate food was available to them at all times with supplemental fresh fruits and vegetables in regular intervals. The experiments were approved by the local authorities in charge (Regierungspräsidium Tübingen, licenses ZP 1/15 and ZP 02/20G) and conducted in accordance with German and European law and the Guidelines for the Care and Use of Laboratory Animals of the National Institutes of Health.

Both monkeys were implanted with titanium head posts and trained to perform behavioral tasks to receive fluid rewards. When training was deemed completed, they were implanted with a recording chamber guided by individual anatomical magnetic resonance images (MRI) and stereotaxic coordinates. The recording chambers were centered over the principal sulcus, anterior to the frontal eye fields, on the left hemisphere (monkey 1, first session, and monkey 2, both sessions), as well as the right hemisphere (monkey 1, second session). Implantation surgeries were performed under general anesthesia using aseptic techniques.

During the experiments, monkeys were head-restrained and seated in primate chairs within darkened chambers. They viewed stimuli on a 15-inch monitor with a 75 Hz refresh rate placed 57 cm in front of them. Behavioral task and stimuli were controlled by CORTEX (NIMH, Bethesda, Maryland, United States of America). Eye movements were tracked continuously and sampled within trials using an infrared eye camera (ISCAN, Woburn, Massachusetts, USA). Responses were registered using a custom-fitted touch-responsive bar in the primate chairs and sampled by CORTEX through a touch monitoring system (Crist Instruments, Hagerstown, Maryland, USA). Fluid reward in a gravity dispenser were controlled by a solenoid valve system (Crist Instruments, Hagerstown, Maryland, USA) connected to CORTEX.

## Methods details

### Sequential numerical decision-making task

The monkeys were trained on a numerical decision task (**Fig 1A**) with stimuli presented sequentially. Each trial started when monkeys grabbed the response bar and fixated the central fixation spot within a gray circle. After 0.5 s of fixation, the first dot display is shown for 0.5 s, to be remembered through a delay of 1 s and compared against a second numerosity which is displayed next for 0.5 s. After another delay of 1 s during which monkeys could decide whether the first and second dot displays had the same number of dots, or different. A colored square would follow (<0.8 s) and instruct them how to respond, red rule: release if different, hold if same, or blue rule: release if same, hold if different. Monkeys received a liquid reward for pairing the decision and rule with the correct motor response. Erroneous trials ended in a time-out for 1 to 2 s. The monkeys were required to fixate the center of the screen throughout the trial and hold the response bar until a response was appropriate.

The numerosity stimuli depicted as black dots on a background gray circle were newly generated before every session using custom MATLAB scripts. Each trial was specified with a certain first number (1, 3, or 9 dots), second number (1, 3, or 9 dots), stimulus protocol (standard or control dot array), decision, and rule cue (red or blue). The stimulus protocols were generated for lower level visual features in the numerosity stimuli. “Standard” format consisted of circles with randomly chosen radii and spacing. “Control” stimuli ensured that numerosity 1, 3, or 9 had the same total colored area and density, by controlling the dot radii and spacing. All these factors were counter-balanced against the decision (same or different) resulting in 48 unique conditions.

As the monkeys performed the task, we monitored their eye position and their hold of the response bar. In total, we recorded 242 sessions of behavior, 110 sessions from Monkey 1 and 132 sessions from Monkey 2. The monkeys’ comparison performance was recorded for each session. We compared their performance to the rule using *signrank* function. Their discrimination of the numerosity was collected as a function of numerical distance between the 2 numerosities. The monkeys’ reaction time was recorded for the release trials, when the numerical decision matched the rule, for example, different numbers followed by a red rule. Release time was specified as the time from the rule cue onset to the release of the bar. We used a 3-way ANOVA to test discrimination behavior against an alpha of 0.01.

### Electrophysiological recordings from the dorsolateral prefrontal cortex

Both subjects were implanted with a titanium head bolt and circular recording chambers over the areas of interest. The positions for implantation were determined from MRI scans to target the dorsal bank of the principal sulcus. Neurons were recorded using 3 to 12 glass-coated titanium electrodes simultaneously. The electrodes were advanced using custom-built manual micro drives or modified electronic drives (NAN drives). We recorded single neurons encountered in every session and did not pre-select them. Waveforms were digitized (Plexon) and stored for offline spike sorting (Offline Sorter, Plexon) and analyses. In total, 691 units were recorded and sorted from both monkeys (Monkey 1: 291 neurons, Monkey 2: 400 neurons).

## Quantification and statistical analysis

All data analysis was performed using MATLAB software. For all effect size and ANOVA analyses, we considered each single unit that displayed a minimum of 0.5 Hz firing rate during the trial (from fixation to rule) and was recorded for a minimum of 30 completed trials. The raw data therefore consisted of a spike table of the form *trials x time* from the start of the fixation period (0 s) to 0.2 s after the rule was presented (at 3.5 s).

### Single-unit analysis of selectivity

As a first step to compare the selectivity of single neurons to the first number, second number, and the monkeys’ decision during the relevant task periods, we performed statistical tests in sliding temporal windows. All the neurons (*n* = 691 neurons) that cleared the minimum trial number and firing rate criteria were analyzed to quantify the influence of trial features on their responses.

### Explained variance analysis

We quantified the influence of the task features on single neurons using an ω^2^-explained variance analysis in a time-resolved manner. The effect of first number, second number, and decision were the primary features we tested, besides stimulus protocol. The ω^2^-explained variance is a measure of effect size that is derived from an ANOVA, to analyze the variance in the trial-by-trial firing rates that is explained by the factor of interest.


$$\omega^{2}=100*(\frac{{SS}_{effect}-({df}_{effect})({MS}_{error})}{{MS}_{error}}+{SS}_{total})$$

To analyze the temporal evolution of signals, we used a *boxcar* window of 200 ms slid by 20 ms along the trial to collect the spikes within the window. We subsampled the trials 100 times to result in equal number of trials per level for the factor. The final PEV used for neuron selection and quantification is the mean of those 100 subsamples. To test for significant encoding of each trial factor, we tested the true PEV against the null distribution derived from 1,000 permutations with shuffled factor labels. We used the 99th percentile of the null distribution to determine when the true PEV is above threshold for 10 consecutive windows. Neurons that crossed this threshold were considered to encode that factor. The end of the first window where the true PEV crossed the threshold was considered the latency of selectivity. The firing rates during the longest consecutive significant bins were used to determine the neuron’s preferred factor level. The mode of the distribution of factor levels that elicited higher firing rates in those bins was calculated as the preferred numerosity. We tested the population encoding of trial features by directly testing the distribution of 100 PEVs for one factor against those calculated for another using Wilcoxon sign-rank test (*signrank*). For the normalized PEV plots, the PEVs for each task factor were normalized to the maximum PEV per factor per neuron.

Neurons showed sequential selectivity to first and second numerosity, as well as decision. To confirm that the sequential selectivity was not just an artifact of ordering, we split the trials into 2 parts, calculated PEV from the training half, and sorted them by the peak value. We ordered the test half by the indices from the training half. The sequential activity remained and so did the correlation between the peak information bins. As a secondary validation, we also calculated the PEV with 100 ms windows moved in 25 ms windows (99th percentile, 5 consecutive windows) and found sequences and correlated peak bins for all the task features. Secondary analysis of the explained variance was done after normalizing the PEV for each neuron and factor against the maximum for that neuron in that factor. To compare PEV values for features, we calculated Spearman’s correlation, evaluated at *P* < 0.01.

Tuning curves were calculated for each neuron by averaging the neuron’s responses to different numerosities in the number presentation period. For the first numerosity, we used the first presentation period of 0.5 s offset by 100 ms to accommodate the visual latency of responses in dlPFC. For the second numerosity, we also used a 0.5 s period beginning at 2.1 s after the start of trial. These response profiles were cross-correlated across presentation periods for the same neurons for the first numerosity, the second numerosity as well as cross-sequence. Single neuronal response profiles were normalized to the preferred numerosity for plotting. Shuffle predictors were calculated by creating a null distribution of CCs by randomly shuffling the association between firing rates and numerosities (1,000 reps). Plotted are means plus 3 standard deviations (across neurons and time windows).

### Neural decoding analysis

For the decoding analyses, we treated the neurons recorded over separate sessions as a pseudo-population. To ensure the same population for all features and comparable training, we selected neurons with at least 20 trials per level of a task factor (examples, 20 trials each with 1 dot as first numerosity, 3 dots as first numerosity and so on) for the task factors, stimulus protocol, first number, second number, decision, rule, action (hold or release). This check gave us a pseudo-population of 537 neurons. We trained SVMs as decoders, as implemented by the MATLAB function *fitcecoc*. We ran 20 runs of decoding for each assessment where we drew 10 training trials for each level and tested the decoders on 10 unseen test trials. We did this resampling for each run to get an average estimate from the pseudo-population [63,64].

We collected firing rates in discrete windows of 50 ms and smoothed by a Gaussian kernel of σ = 1. Performance of the decoders were quantified as the percent of held-out test trials correctly classified as the target factor, for example, first numerosity 1, 3, or 9. Mean decoding performance for target features was the mean of decoder performance from 20 runs of decoding, each time drawing a set of training and hold-out test trials. Decoder performance was tested by conducting the decoding with 1,000 permutations of shuffled trial labels. Significance was evaluated at *P* < 0.01. For trial features with more than 2 levels, we also calculated classification probability to estimate the relationships between factor levels. We did these within time bins of statistically significant decoding accuracy, or equivalent (in case of nonsignificant cross-order number decoding).

To test the temporal dynamics in the neural population, we conducted cross-temporal decoding by training a decoder in one time bin and testing it on another. We tested these statistically by conducting 1,000 permutations of each decoder by shuffling trial labels but maintaining the trial’s temporal structure. To statistically determine the temporal stability, we compared the decoding accuracy on the time diagonal against the accuracy off-diagonal by testing those distributions using Wilcoxon sign-rank test (*signrank*).

To test for consistency in the population for numerosity coding, we performed cross-order decoding of number such that we trained the SVM to classify the first number (classify as 1, 3, or 9), and tested it with test trials with second number as 1, 3, or 9. We also performed the reverse, by training classifiers on the second number and testing it to predict the first number. We repeated these analyses on subsampled trials 20 times and calculated a null distribution using 1,000 permutations (*P* < 0.01) of training trials.

To directly compare sequence decoding against decision decoding, we extracted a subpopulation of 368 neurons with at least 5 trials per sequence of numbers (11, 13, 19, 31, and so on). We tested this pseudo-population at classifying sequence and decision, using 3 training trials per sequence and 10 training trials per decision, testing them on 10 held-out test trials. We repeated the decoding 20 times, sampling trials at random and tested it against the null distribution (*P* < 0.01) of 1,000 decoders trained on shuffled training labels.

To test for decision generalization, we trained decision classifiers on trials involving only 2 of the number, while leaving trials of the third number out. For example, training the classifier to predict “same” or “different” based on trials involving numerosity 3 and 9, 5 training trials per decision, and tested it on 10 held-out trials where either the first or second numerosity would be 1. We performed this test by leaving out each numerosity in turn, repeated 20 times, and permuted 1,000 times (*P* < 0.01). Scores calculated over significant time bins were collected.

### Subspace analysis

To analyze the lower-dimensional transformations of neural activity, we performed dimensionality reduction analyses [20]. Using the same binned and smoothed firing rates per neuron as for the decoding analyses, we split the trials into 2 halves. We use the training half to perform the dimensionality reduction and projected the activity from the testing half to the axes resulting from the PCA. For the first subspace, we sorted the trials into 3 (1, 3, or 9) number bins and 2 (same or different) decision bins, resulting in 6 conditions (each first num-decision pair).

$$X=\left[\begin{array}{c} r(N_{1,S})-\bar{r}\\ \vdots \\ r(N_{1stnum,Dec})-\bar{r} \end{array}\right]$$

Where *r*(*N*_1*st num*,*Dec*_) is the mean population vector across trials for the condition corresponding to first number 1 and decision Same, and $\bar{r}$ is the mean population vector across the 6 conditions. We similarly created a separate subspace for the second number with decision. The principal components of this matrix were identified by decomposing the covariance matrix C of X using singular value decomposition (as implemented by pca.m in MATLAB): C = PDPT, in which each column of P is an eigenvector of C and D is a diagonal matrix of corresponding eigenvalues. We constructed a reduced (*K* = 3)-dimensional space whose axes correspond to the first *K* eigenvectors of *C* (i.e., columns of *P*, *P*_*K*_). These first 3 eigenvectors explained an average of 65% of the variance in the mean population response across all examined time points. We then projected the population vector for a given condition into this reduced dimensionality space: $z_{K}=P_{K}^{T}(r(N_{1st,Dec})-\bar{r})$, in which *z*_*K*_ is the new coordinate along axis *K* in the reduced dimensionality space.

We calculated the cosine of the angle between the 2 planes as follows:

$$\cos\left(\theta \right)=(v_{S1}\times v_{S2})\times (v_{D1}\times v_{D2}).$$

Where the vectors defining the plane for same decision are *v*_*S*1_ and *v*_*S*2_, and those for the different decision are *v*_*D*1_ and *v*_*D*2_. We used a bootstrapping procedure to calculate cos(*θ*) in 1,000 iterations with different samples of training and test trials according to the condition each time across neurons.

## Supporting information

S1 Fig Effect of stimulus protocol for numerosity on behavior. (A) Example display for visual controls used in our task. (B) Performance of monkeys 1 and 2 calculated for each session plotted as single dots with mean ± SEM. (C) Reaction time calculated as time from rule cue to bar release for trials where decision matched motor rule. Box plots show median with boxes spanning 25–75 percentile of data. The data underlying this and all other figures is available at https://doi.org/10.6084/m9.figshare.25046987.
(DOCX)

S2 Fig Mixed selectivity for number and decision. Venn diagram showing the number of neurons selective for first number, second number, and decision as well as the overlap between them. Selective cells chosen from significant omega-squared explained variance than 99th percentile of shuffled data (*P* < 0.01). The data underlying this and all other figures is available at https://doi.org/10.6084/m9.figshare.25046987.
(DOCX)

S3 Fig Effect size for number and decision are correlated only for neurons selective to second num. (A) Tuning curves averaged over selective neurons colored by preference to first number (*left panel*) or second number (*right panel*) collected in the same presentation period. (B) The same neurons’ tuning curves from responses collected in the other presentation periods. (C) Normalized peak effect size for number is plotted against peak effect size for decision for neurons selective for first number, second number, respectively. Significant and strong correlation is exhibited by neurons selective for second number. The data underlying this and all other figures is available at https://doi.org/10.6084/m9.figshare.25046987.
(DOCX)

## Abbreviations

dlPFC: dorsolateral prefrontal cortex
PEV: proportion of explained variance
PFC: prefrontal cortex
SVM: support vector machine
