# Supplementary material for: Sequential neuronal processing of number values, abstract decision, and action in the primate prefrontal cortex
Source: PLoS Biol. 2024 Feb 16;22(2):e3002520. doi: 10.1371/journal.pbio.3002520 (PMC10871863; doi:10.1371/journal.pbio.3002520)
Supplement: S1 Fig — (A) Example display for visual controls used in our task. (B) Performance of monkeys 1 and 2 calculated for each session plotted as single dots with mean ± SEM. (C) Reaction time calculated as time from rule cue to bar release for trials where decision matched motor rule. Box plots show median with boxes spanning 25–75 percentile of data. The data underlying this and all other figures is available at https://doi.org/10.6084/m9.figshare.25046987. (DOCX) [file pbio.3002520.s001.docx]

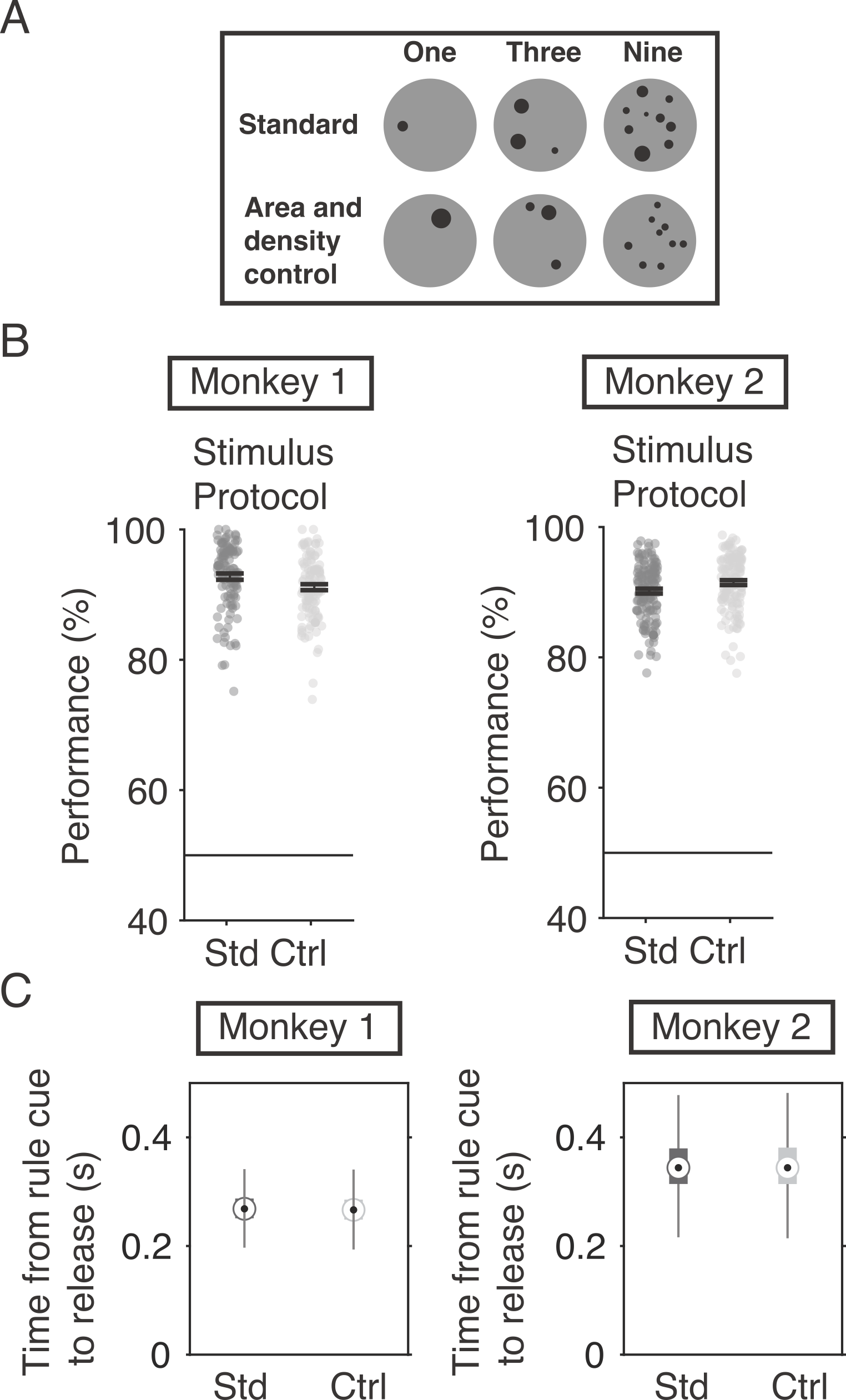


**S1 Fig: Effect of stimulus protocol for numerosity on behavior**

(**A**) Example display for visual controls used in our task.

(**B**)Performance of monkeys 1 and 2 calculated for each session plotted as single dots with mean ± SEM.

(**C**) Reaction time calculated as time from rule cue to bar release for trials where decision matched motor rule. Box plots show median with boxes spanning 25-75 percentile of data.
