# Supplementary material for: Sequential neuronal processing of number values, abstract decision, and action in the primate prefrontal cortex
Source: PLoS Biol. 2024 Feb 16;22(2):e3002520. doi: 10.1371/journal.pbio.3002520 (PMC10871863; doi:10.1371/journal.pbio.3002520)
Supplement: S2 Fig — Venn diagram showing the number of neurons selective for first number, second number, and decision as well as the overlap between them. Selective cells chosen from significant omega-squared explained variance than 99th percentile of shuffled data (P < 0.01). The data underlying this and all other figures is available at https://doi.org/10.6084/m9.figshare.25046987. (DOCX) [file pbio.3002520.s002.docx]

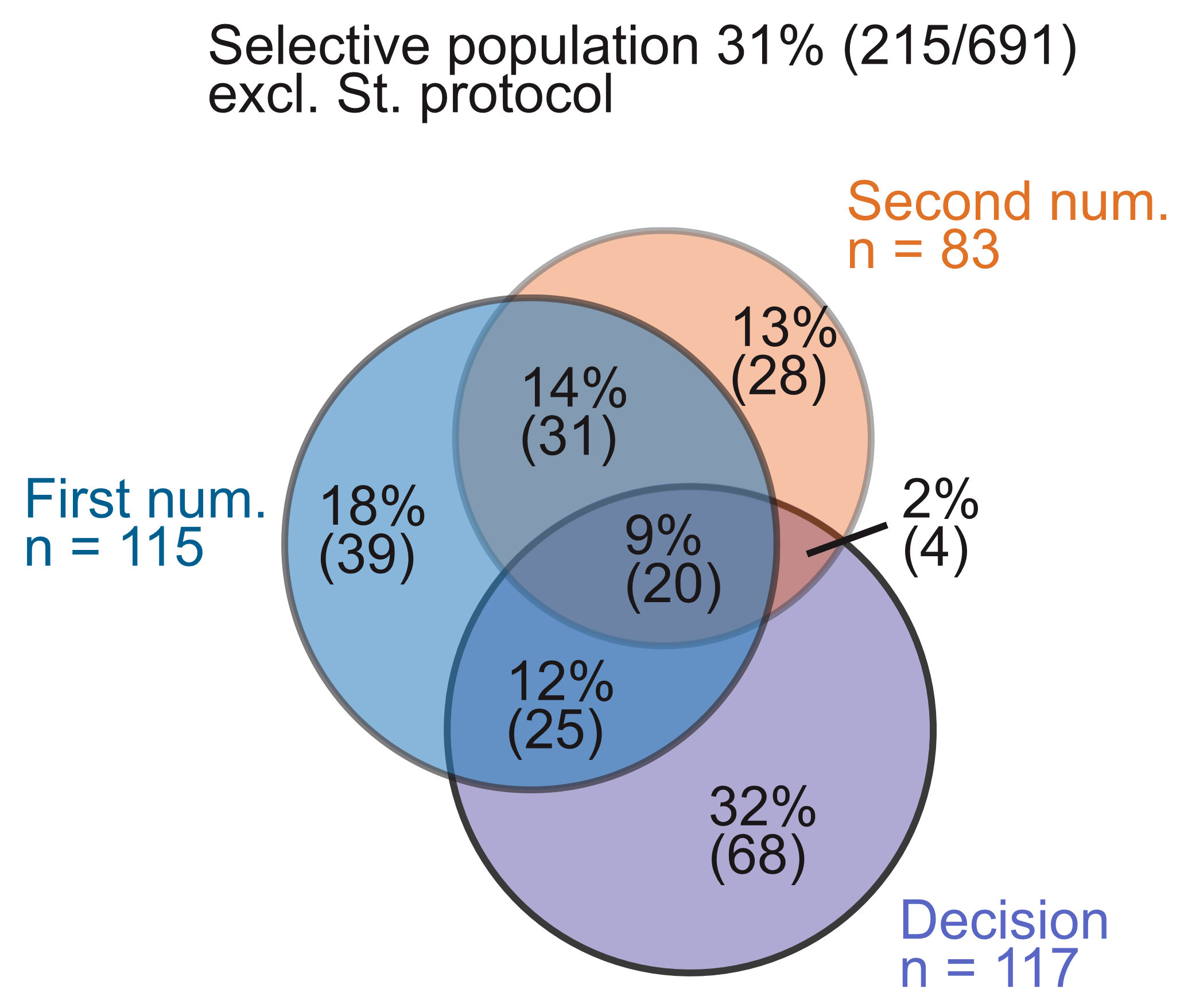


**S2 Fig: Mixed selectivity for number and decision**

Venn diagram showing the number of neurons selective for first number, second number and decision as well as the overlap between them. Selective cells chosen from significant omega-squared explained variance than 99^th^ percentile of shuffled data (P<0.01).
