# Supplementary material for: Sequential neuronal processing of number values, abstract decision, and action in the primate prefrontal cortex
Source: PLoS Biol. 2024 Feb 16;22(2):e3002520. doi: 10.1371/journal.pbio.3002520 (PMC10871863; doi:10.1371/journal.pbio.3002520)
Supplement: S3 Fig — (A) Tuning curves averaged over selective neurons colored by preference to first number (left panel) or second number (right panel) collected in the same presentation period. (B) The same neurons’ tuning curves from responses collected in the other presentation periods. (C) Normalized peak effect size for number is plotted against peak effect size for decision for neurons selective for first number, second number, respectively. Significant and strong correlation is exhibited by neurons selective for second number. The data underlying this and all other figures is available at https://doi.org/10.6084/m9.figshare.25046987. (DOCX) [file pbio.3002520.s003.docx]

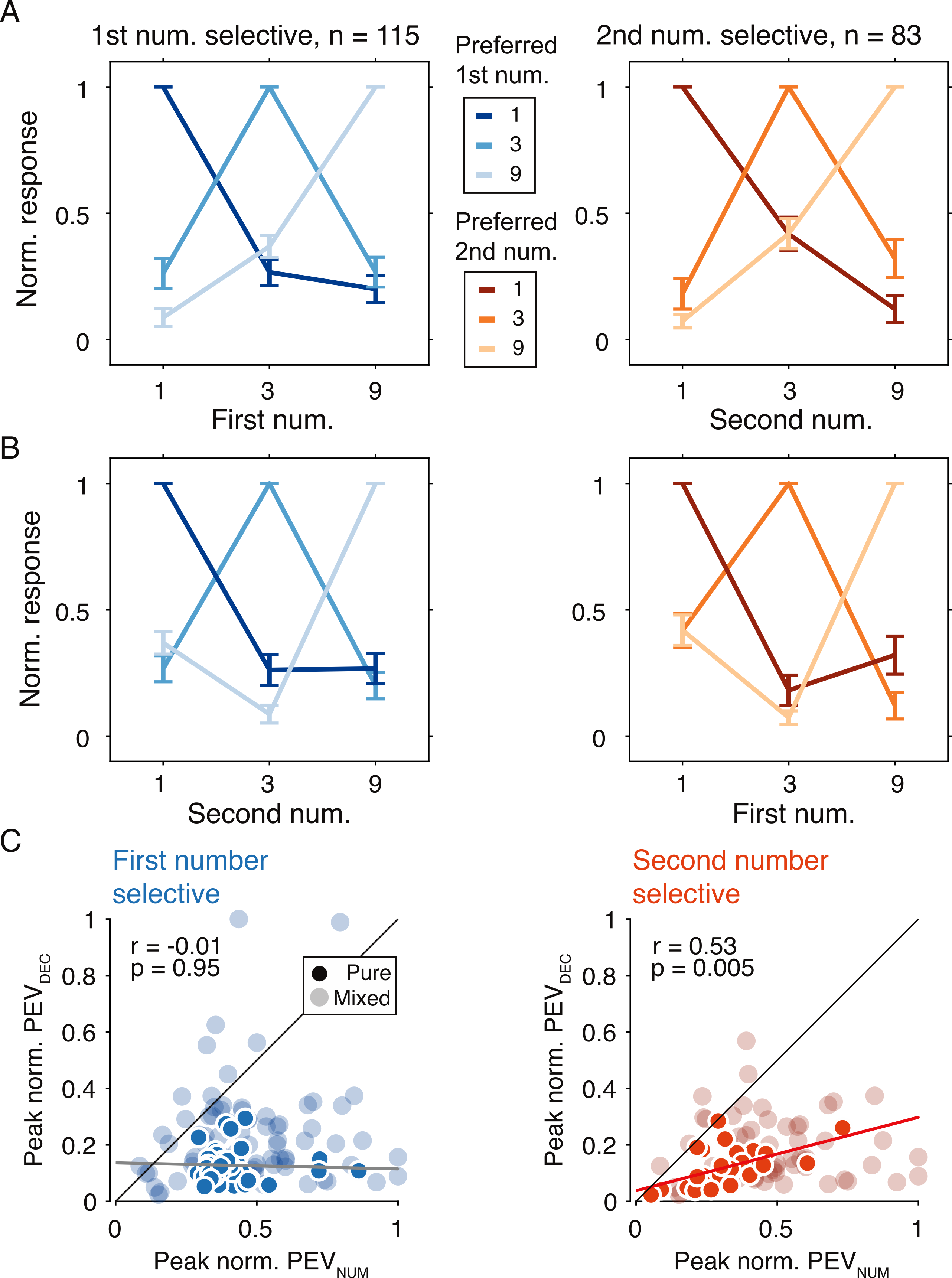


**S3 Fig: Effect size for number and decision are correlated only for neurons selective to 2nd number.**

(**A**) Tuning curves averaged over selective neurons colored by preference to first number (*left panel*) or second number (*right panel*) collected in the same presentation period.

(**B**) The same neurons’ tuning curves from responses collected in the other presentation periods.

(**C**) Normalized peak effect size for number is plotted against peak effect size for decision for neurons selective for first number, second number, respectively. Significant and strong correlation is exhibited by neurons selective for second number.
